# Life History Consequences of the Facultative Expression of a Dispersal Life Stage in the Phoretic Bulb Mite (*Rhizoglyphus robini*)

**DOI:** 10.1371/journal.pone.0136872

**Published:** 2015-09-01

**Authors:** Jacques A. Deere, Tim Coulson, Isabel M. Smallegange

**Affiliations:** 1 Department of Zoology, University of Oxford, Oxford, United Kingdom; 2 Institute for Biodiversity and Ecosystem Dynamics (IBED), University of Amsterdam, Amsterdam, The Netherlands; Estación Experimental de Zonas Áridas (CSIC), SPAIN

## Abstract

Life history traits play an important role in population dynamics and correlate, both positively and negatively, with dispersal in a wide range of taxa. Most invertebrate studies on trade-offs between life history traits and dispersal have focused on dispersal via flight, yet much less is known about how life history trade-offs influence species that disperse by other means. In this study, we identify effects of investing in dispersal morphology (dispersal expression) on life history traits in the male dimorphic bulb mite (*Rhizoglyphus robini*). This species has a facultative juvenile life stage (deutonymph) during which individuals can disperse by phoresy. Further, adult males are either fighters (which kill other mites) or benign scramblers. Here, in an experiment, we investigate the effects of investing in dispersal on size at maturity, sex and male morph ratio, and female lifetime reproductive success. We show that life history traits correlate negatively with the expression of the dispersal stage. Remarkably, all males that expressed the dispersal life stage developed into competitive fighters and none into scramblers. This suggests that alternative, male reproductive strategies and dispersal should not be viewed in isolation but considered concurrently.

## Introduction

Life history traits, such as age and size at maturity, play a crucial role in population dynamics as they directly influence reproduction and survival. Importantly, their evolution is constrained by trade-offs [1–3]. Dispersal effects have been shown to correlate with life history traits, both positively and negatively, in a wide range of taxa [1, 2, 4–8] (but see 3]). Identifying which life-history traits co-vary with dispersal-related traits will allow a better understanding of the evolutionary dynamics of dispersal [7, 9].

The literature on life history trade-offs and dispersal is especially abundant for terrestrial invertebrates, with a strong focus on fecundity and survival as the traits of interest. Most invertebrate studies have focused on dispersal via flight, with varying results in wing-monomorphic and wing-polymorphic species [5, 9, 10, 11]. Studies on wing-monomorphic species have shown that fecundity is higher in dispersive than in sedentary individuals [10, 12, 13]. It has been suggested that this outcome is due to physiological effects: in the Glanville fritillary butterfly *Melitaea cinxia* [13] for example, high metabolic performance resulted in high dispersal and oviposition rates whereas increased juvenile hormone levels enhanced reproduction after long-duration flight in the grasshopper *Melanoplus sanguinipes* [12]. In contrast, the majority of studies on wing-polymorphic species has reported negative correlations between dispersal capability and fecundity [14] (but note that several exceptions exist: see review by [11]). Few studies exist that examine which trade-offs are involved when dispersal occurs via means other than flight such as phoresy, ballooning, or walking, and their results vary [15–17]. For example, in the two-spotted spider mite (*Tetranychus urticae*), no trade-off was found between dispersal by ballooning and fecundity [15], whereas fecundity was reduced if mites dispersed by walking [16]. In addition, when looking at other life-history traits, again in *Tetranychus urticae*, the traits can be correlated to different dispersal modes [18] or not [19]. This suggests that different dispersal modes within the same species may or may not correlate with the same, or different, life history traits. Insights from all these studies can be summarised into the following points. Firstly, life history traits can relate both positively and negatively to dispersal. Secondly, the type of association can depend not only on the species but also on the mode of dispersal. Finally, there are very few studies on trade-offs between non-flight dispersal and life-history traits. These observations highlight the need to investigate dispersal-induced life history trade-offs further, both in a wider range of taxa and of dispersal-related traits.

Here we aim to investigate if trade-offs exist between investing in dispersal (which we refer to as dispersal expression) and life history traits in the bulb mite (*Rhizoglyphus robini*, Acaridae), a species that disperses by phoresy. Dispersal occurs when juveniles develop into the (facultative) deutonymph stage in response to unfavourable environmental conditions. The development of this dispersal stage requires energetic investment and in other taxa energetic investment in dispersal morphology has been shown to be costly [14]. When juveniles develop into this stage, a sucker plate on their ventral side allows them to attach to invertebrate hosts, such as beetles. The bulb mite is also male dimorphic; males are either fighters, which kill other mites with their thickened third pair of legs, or scramblers, which do not have this modification and are defenceless [20]. Male morph determination is complex: fighters emerge from larger final instars [21], and, recently, Leigh and Smallegange [22] suggest that early male ontogeny, as well as environmental quality, also plays a role in male morph determination. Whether male morph and dispersal expression are related is yet unknown. Here, in an experiment, we investigate for both sexes and both male morphs, the effect of investing in the dispersal stage on key life-history traits; size at maturity and, in females, lifetime reproductive success. Life history traits such as size at maturity have been shown to be a good indicator of individual fitness in many species. For example, individuals may show a higher fitness if they reach a certain size [23, 24]. Size at maturity is also shown to be indicative of morph determination [25, 26], with one morph often having a higher fitness than the other. Additionally, lifetime reproductive success has been shown to be influenced by individual size and environmental conditions [27, 28]. Furthermore, the shape of the distribution of body sizes within a population, influences population dynamics (see [29–31]).

To this end, we compare life history traits of individuals that did not develop into a deutonymph (non-dispersers) with individuals that did develop into a deutonymph (dispersers) during their development. Furthermore, we tested whether individuals compensate for lost growth associated with deutonymph development [32, 33], for example by increasing their growth rate in a subsequent life stage. Because we could not directly control dispersal expression, our results should be interpreted as correlational rather than causational evidence for dispersal costs. We conclude by discussing possible consequences of dispersal expression on these life-history traits and highlight how these consequences may affect the evolution of life-history in dispersal capable species.

## Methods

### Study species

**Fig 1:**
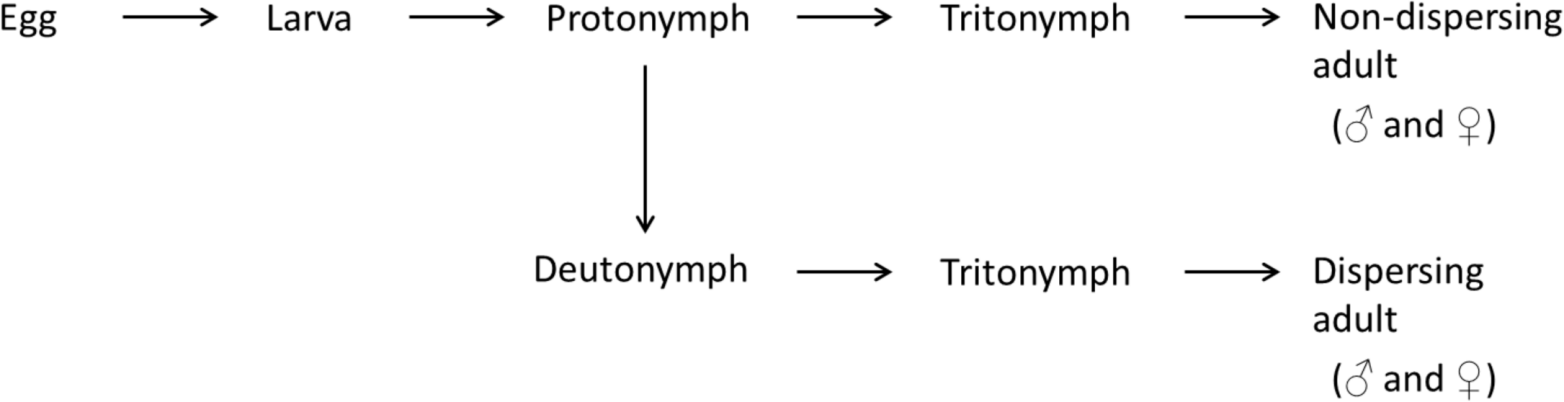
Life cycle of the bulb mite. The life cycle has six life stages; the deutonymph stage is the facultative dispersal stage that develops under unfavourable conditions. Male morph determination is dependent on the size of the tritonymph stage. Here, we found that adult males that had expressed the dispersal stage all matured as fighters (see *Results*).

The bulb mite is a cosmopolitan pest species with a broad host range [34]. Its life cycle consists of six life stages: egg, larva, protonymph, deutonymph (non-feeding and facultative), tritonymph and adult (Fig 1). Deutonymph expression depends on environmental conditions including temperature, humidity, food quality or a combination thereof (see review by [34]) and increases as environments deteriorate (e.g. decrease in food quality or decrease in temperature and humidity). To our knowledge, population density does not play a role in deutonymph expression. Longevity and generation time of both sexes is dependent on temperature and food quality. Longevity can be as short as 14 days (fed on garlic at 35°C) or as long as 73 days (fed on peanuts at 27°C) [34], while generation time can be as short as 12 days when fed yeast at 24°C [35] or as long as 56 days (fed on garlic at 16°C) [34]. Reproduction is strictly sexual [34].

### Data collection

#### Stock cultures

Data were collected between August 2012 and May 2013. The mites were taken from stock cultures that were collected in December 2010 from private flower bulb storage rooms (with permission from Koppert Biological Systems) in the Netherlands. No local or government authority was involved and collection and transport of the mites was in line with Dutch law on the use of animals in ecological studies. The stock cultures were maintained on oats, which produce viable populations and induce deutonymph expression (personal observation). In comparison, mites maintained on yeast under the same temperature and humidity do not produce deutonymphs. The difference in protein content between yeast and oats (yeast has higher protein content than oats), and consequently the reduction in food quality of the oats environment, is the likely cause of increased deutonymph expression. The cultures were maintained as described in [35].

#### General outline of data collection

To build our dataset, we first reared individually isolated mites from eggs to adult and documented their life-history trajectory. However, this initial dataset contained only a very small proportion of individuals that expressed the deutonymph stage (3 from 132 individuals isolated as eggs), with which we could not statistically compare the life history trajectories of individuals that expressed the deutonymph stage and those that did not. We therefore supplemented our dataset by documenting the life history trajectories of mites that were individually isolated from the stock culture as deutonymphs and protonymphs (Fig 1). By doing so we assume that (i) development during the egg to protonymph stages did not differ between individuals that did not develop into the deutonymph stage and those that did, and (ii) any differences in life history traits between dispersers and non-dispersers are due to deutonymph expression and occur after this point in development. We cannot test the second assumption, but tested the first one by comparing the demographic trajectories of non-dispersing individuals (i.e. individuals that do not express the deutonymph stage) that were either isolated as eggs (initial dataset) or as protonymphs (supplemented data). This comparison revealed no significant difference in growth and survival between non-dispersing individuals isolated from the stock culture as eggs or as protonymphs (**S1 Table**). We therefore combined the initial and supplemented datasets (further details of data collection are given below). In the combined dataset, individuals that had been a deutonymph were coded as 1 (deutonymph stage present) and those that had not were coded as 0 (deutonymph stage absent).

#### Initial dataset (individuals isolated as eggs)

A total of 27 females were isolated from the stock cultures over three consecutive, replicate time periods: 15 in the first period, 6 in the second and 6 in the third. These females were allowed to lay eggs for 3–8 days. A total of 132 eggs were collected over the three replicates time periods: replicate one, 51 eggs collected over 7 days; replicate two, 54 eggs collected over 8 days; and replicate three, 27 eggs collected over 3 days. Eggs were collected daily from each female, and individually isolated into single tubes with *ad libitum* oats. The tubes were kept in an unlit incubator at 24°C and >70% relative humidity. Until maturation, each individual was photographed daily using a Lumenera Infinity 3.1 camera (Lumenera Corporation, Ottawa, 22 Ontario, Canada) connected to a Meiji 20 EMZ-8TRD (10–45x) stereomicroscope and its length (without mouthparts) measured to the nearest 0.001 mm using Infinity Analyze Imaging Software (Lumenera Corp.). Henceforth body length is referred to as size. Some individuals died before reaching maturity (replicate time period 1, *n* = 25; replicate time period 2, *n* = 33; replicate time period 3, *n* = 14), and of the 132 eggs only 3 subsequently developed into deutonymphs. Mature females were mated with randomly chosen virgin males (from either morph), which were placed in the same tube for the duration of the female’s lifetime. Eggs were counted daily until the female died.

#### Supplemental data (individuals isolation as protonymphs)

Protonymphs were individually isolated from the stock cultures over two replicate time periods. For the two replicates, each over the course of 5 days, 25 protonymphs were isolated (5 protonymphs per day). The isolated individuals were maintained under the same conditions, and monitored in the same way, as individuals isolated as eggs (see above). Twenty-four of the 50 individuals died before reaching maturity (replicate time period 1, *n* = 18; replicate time period 2, *n* = 6).

#### Supplemental data (individuals isolation as deutonymphs)

Deutonymphs were individually isolated from the stock cultures over three replicate time periods. For the first two replicates, each over the course of 5 days, 26 deutonymphs were isolated (day 1–4, 5 deutonymphs per day; day 5, 6 deutonymphs). Thirty deutonymphs were isolated over 5 days in the third replicate (6 individuals per day). The isolated individuals were maintained under the same conditions, and monitored in the same way, as individuals isolated as eggs (see above). Thirty-seven of the 82 individuals died before reaching maturity (replicate time period 1, *n* = 14; replicate time period 2, *n* = 14; replicate time period 3, *n* = 19).

### Statistical analyses

Five different analyses were conducted. Firstly, to determine whether the sex ratio, and the ratio of male fighters to male scramblers differed between dispersers and non-dispersers, we tested for equality of proportions by conducting a 2-sample test using the function *prop*.*test*. Secondly, we used the supplemental data of individuals isolated as deutonymphs to test the effect of time spent in the deutonymph stage (deutonymph duration), deutonymph size, their interaction, sex and replicate time period on size at maturity of dispersers using a generalised linear model (GLM) with a Gaussian distribution. Thirdly, we used the combined dataset (including the life history trajectories of dispersers and non-dispersers), to assess the relationship between the explanatory variables sex, deutonymph expression, their interaction and replicate time period and the response variable size at maturity using a GLM with a Gaussian distribution. Fourthly, to assess whether dispersers compensate for reduced growth as a result of developing into a deutonymph, we used the combined dataset to test, for each sex, whether total growth (mm) and the standardised growth rate (d^-1^; non-dispersers, growth per day per protonymph size; dispersers, growth per day per deutonymph size) during the tritonymph stage differed between non-dispersers and dispersers. For each compensatory growth analysis, we used a GLM with a Gaussian distribution; deutonymph expression and replicate time period were the explanatory variables. We predict that if dispersers compensate for reduced growth, total growth for dispersers and non-dispersers during the tritonymph stage would be similar, and the standardised growth rate during the tritonymph stage in dispersers would be higher than that in non-dispersers. Finally, using the combined dataset, we applied a GLM with a Gaussian distribution to test the relationship between the explanatory variables deutonymph expression, female lifespan, their two-way interaction and replicate time period, and the response variable female lifetime reproductive success. Because the combined dataset does not include information on the life history trajectory of mites from egg to deutonymph stage, we could not analyse effects of dispersal expression on age at maturity. All of the statistical analyses were conducted in R using the library lme4 (version 3.0.2) [36].

In each GLM analysis, a model simplification procedure was applied. After fitting the full model, the least significant term from the highest order interaction downwards was identified and removed if the removal resulted in an insignificant increase in deviance (significance was assessed using likelihood ratio tests). The model assumptions of Gaussian errors and homoscedasticity were confirmed by visual inspection of the probability plots and error structures. Parameter estimates (ê) of the explanatory variables in the minimal models are reported in the results section; the parameter estimates were coefficients in the linear regression model, and represent the relationship between an explanatory variable (or an interaction between several explanatory variables) and the response variable. The final models (after model simplification) can be found in the online appendix (**S2 Table).**

## Results

### Effect of deutonymph expression on sex ratio and male morph expression

The sex ratio and the ratio of fighters to scramblers differed between dispersers and non-dispersers. The sex ratio of non-dispersers was not significantly different from 50:50 at 44:56 (female: n = 37; male: n = 48) (*χ* ^*2*^ = 2.353, *df* = 1, *P* = 0.125). The fighter-to-scrambler ratio within non-dispersing males significantly differed from 50:50 at 65:35 (fighter: n = 31; scrambler: n = 17) (*χ* ^*2*^ = 7.042, *df* = 1, *P* = 0.008). In dispersing individuals, the sex ratio of 68:32 was female-biased (female: n = 23; male: n = 11), and significantly different from 50:50 (*χ* ^*2*^ = 7.118, *df* = 1, *P* = 0.008). However, the most striking finding was that no single dispersing male developed into a scrambler, as the fighter-to-scrambler ratio in dispersing males was 100:0 (fighter: n = 11; scrambler: n = 0). Given the male morph ratios, we tested whether the male morph ratio of dispersers, 100:0, and non-dispersers, 65:35, were indeed different. We applied a Fisher’s exact test (no scramblers developed from deutonymphs; n = 0) and found that the male morph ratio differed significantly between dispersers and non-dispersers (*P* = 0.024, Fisher’s exact test). As we did not obtain any data on dispersing scramblers all subsequent analyses that compared the life history traits of dispersers and non-dispersers only included data on fighters and females.

### Effect of deutonymph size and duration on size at maturity of dispersers

Deutonymph size (ê = 1.599±1.019 (s.e.), *t* = 1.570, *P* = 0.129, n = 34), deutonymph duration (ê = 0.035±0.039 (s.e.), *t* = 0.920, *P* = 0.366, n = 34) and replicate time period (ê = 0.005±0.015 (s.e.), *t* = 0.368, *P* = 0.716, n = 34) did not significantly affect the size of dispersers at maturity. However, sex did have a significant effect on size at maturity as dispersing males (0.512mm) matured at a smaller size than dispersing females (0.627mm) (ê = -0.115±0.026 (s.e.), *t* = -4.378, *P* < 0.001, n = 34).

### Effect of deutonymph expression on size at maturity

**Fig 2:**
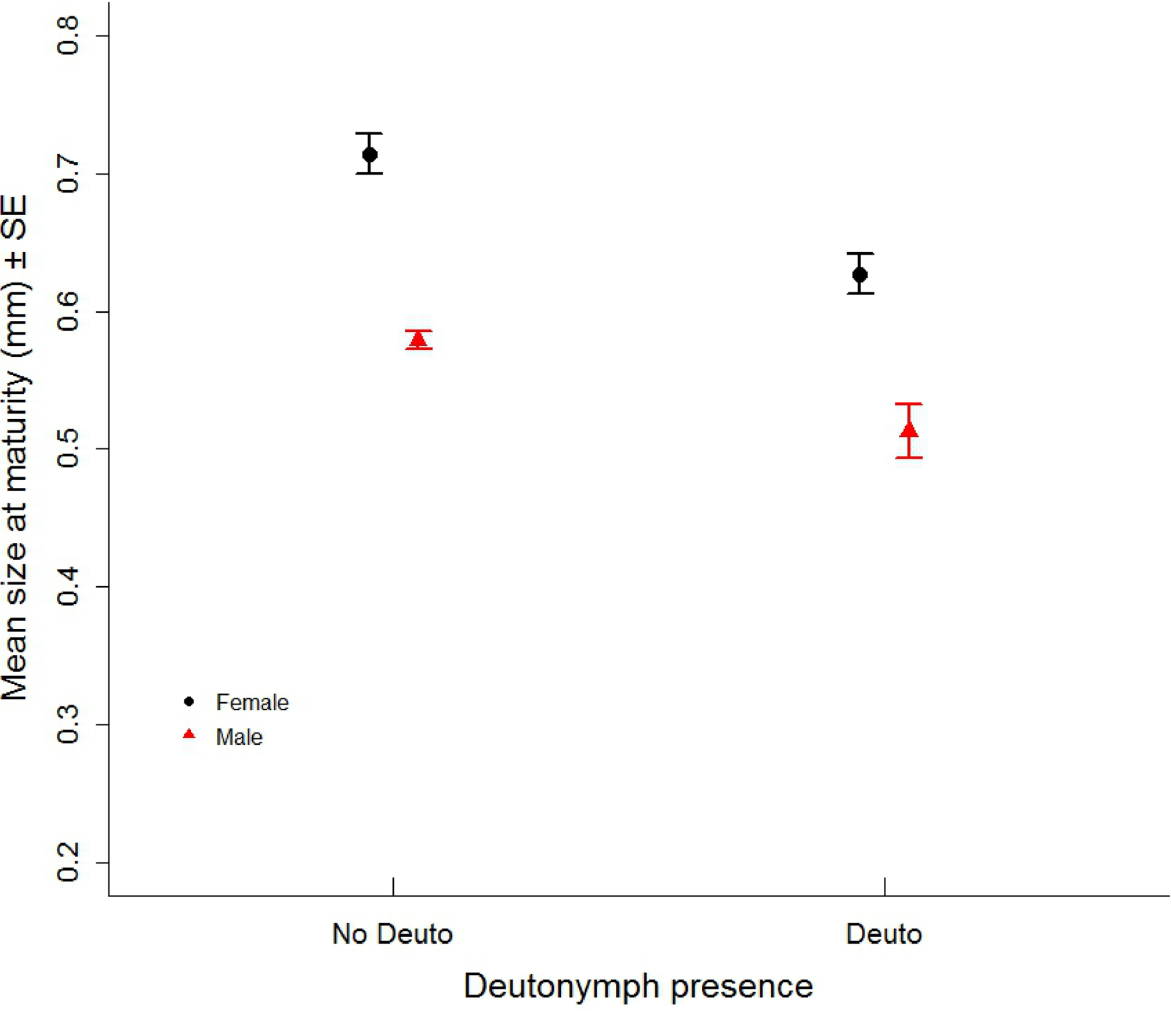
Size at maturity. Size at maturity (mean ± s.e.) for dispersers (Deuto) and non-dispersers (No Deuto), in females (black points and lines) and fighter males (red triangles and lines).

Size at maturity was not significantly affected by the two-way interaction between sex and deutonymph expression (ê = 0.019±0.032 (s.e.), *t* = 0.589, *P* = 0.557). Replicate time period also had no significant effect on size at maturity (Replicate time period: ê = -0.009±0.005 (s.e.), *t* = -1.876, *P* = 0.064). Sex (ê = -0.130±0.014 (s.e.), *t* = -9.114, *P* < 0.001, n = 102) and deutonymph expression (ê = -0.080±0.015 (s.e.), *t* = -5.376, *P* < 0.001, n = 102) did significantly affect size at maturity. Females were larger at maturity (mean ± s.e.: 0.712 ± 0.011, n = 60) than males (0.582 ± 0.014, n = 42), and non-dispersers matured at a larger size (females, 0.712 ± 0.011 (s.e.), n = 37; males, 0.570 ± 0.007 (s.e), n = 31) than dispersers (females, 0.627 ± 0.014 (s.e), n = 23; males, 0.513 ± 0.019 (s.e.), n = 11) (Fig 2).

### Compensatory growth and deutonymph expression

**Fig 3:**
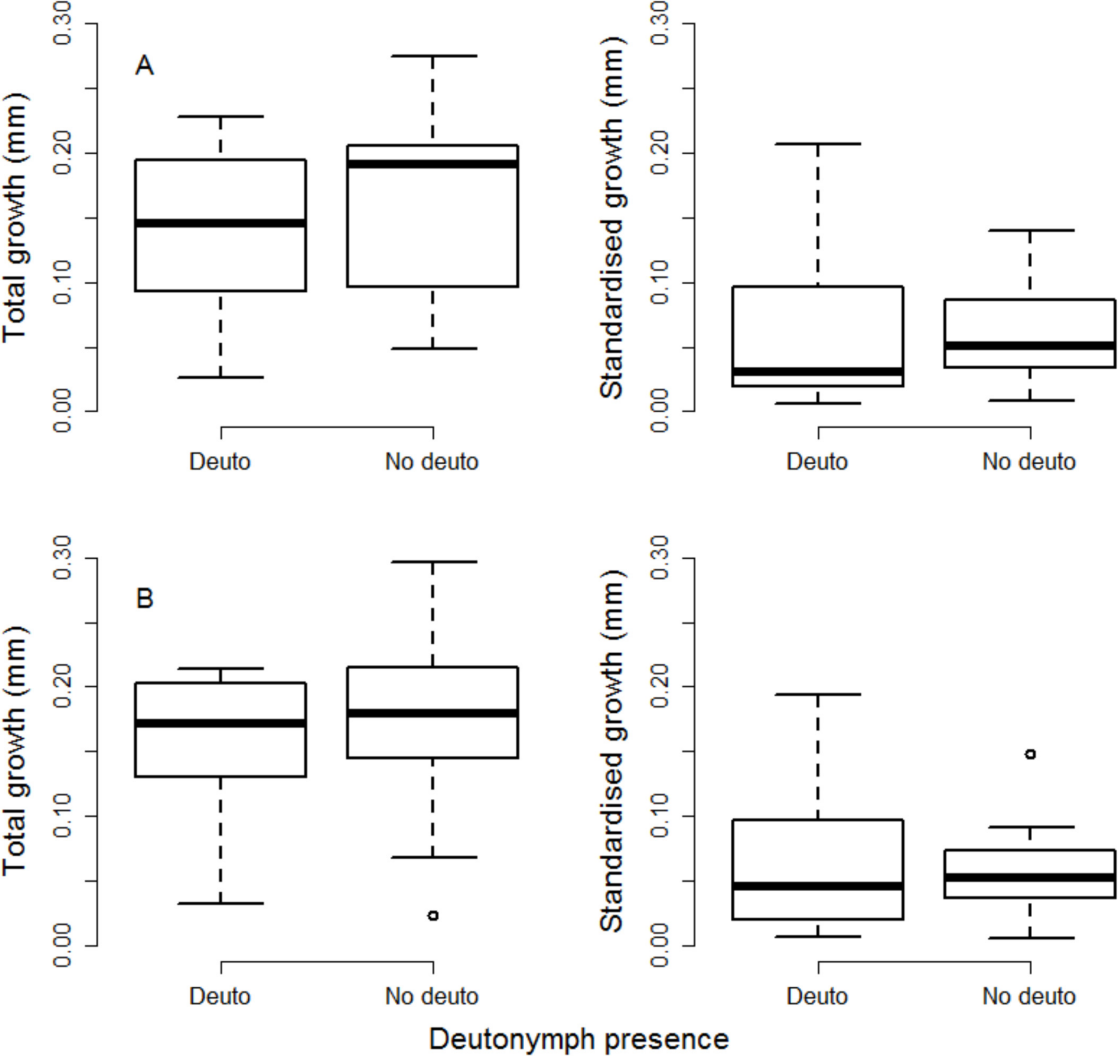
Compensatory growth. Total growth (mm) and standardised growth (mm per day per tritonymph length) during the tritonymph stage, as a function of deutonymph presence (Deuto) or deutonymph absence (No Deuto) during development in females (A) and fighter males (B). Boxes represent upper and lower quartile ranges, middle bands are medians and whiskers represent the extremes. Outliers are shown as points.

Total growth and standardised growth were not significantly different between disperser tritonymphs (that developed from deutonymphs) and non-disperser tritonymphs (that developed from protonymphs) in females (total growth: ê = 0.020 ± 0.024 (s.e.), *t* = 0.869, *P* = 0.391; standardised growth: ê = 0.035 ± 0.032 (s.e.), *t* = 1.094, *P* = 0.281; n = 45) (Fig 3A) or males (total growth: ê = 0.018±0.026 (s.e.), *t* = 0.682, *P* = 0.501; standardised growth: ê = 0.006±0.035 (s.e.), *t* = 0.158, *P* = 0.876; n = 36) (Fig 3B). Total growth and standardised growth were also not significantly different between treatments in females (total growth: ê = -0.005±0.006 (s.e.), *t* = -0.807, *P* = 0.425; standardised growth: ê = 0.005±0.008 (s.e.), *t* = 0.625, *P* = 0.538; n = 45) or males (total growth: ê = 0.003±0.008 (s.e.), *t* = 0.348, *P* = 0.731; standardised growth: ê = 0.006±0.010 (s.e.), *t* = 0.614, *P* = 0.544; n = 36).

### Effect of deutonymph expression on female lifetime egg production

**Fig 4:**
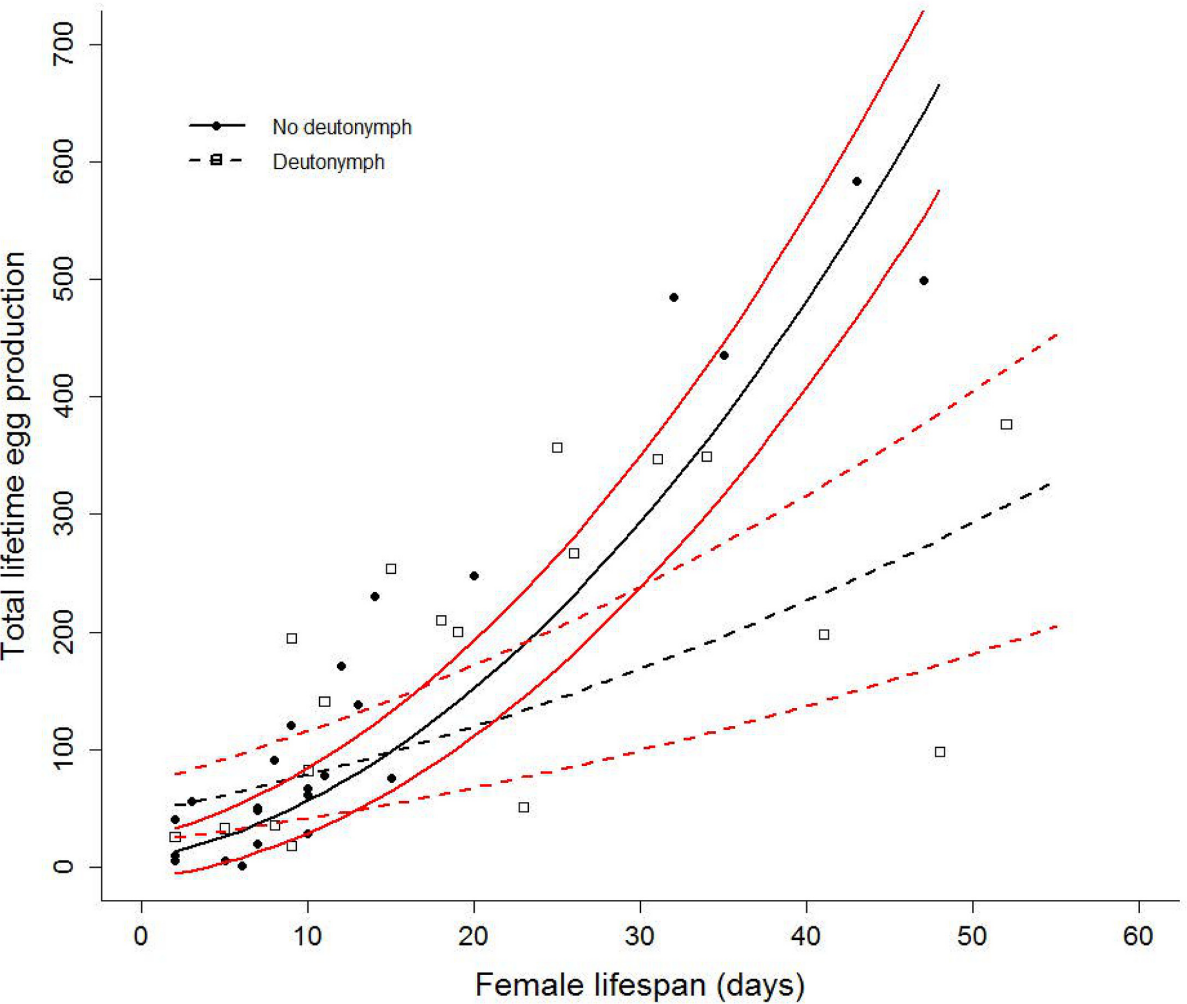
Female lifetime egg production. Lifetime egg production as a function of female lifespan for dispersers (open squares and dashed lines) and non-dispersers (solid points and solid line). Confidence intervals are shown in red; dispersers (dashed red lines), non-dispersers (solid red lines).

There was a significant effect of the interaction between deutonymph expression and female lifespan on female lifetime egg production (ê = -0.275±0.082 (s.e.), *t* = -3.375, *P* < 0.005, n = 42). Female lifetime egg production increased with female lifespan, but the slope of this relationship was steeper for non-dispersing females than it was for dispersing females (Fig 4). As a result, there was no significant difference in lifetime egg production between dispersing and non-dispersing females when female lifespan is low (<30 days). However, as female lifespan increased above 30 days the confidence intervals of the curves did not overlap, and non-dispersing females had a higher lifetime egg production than did dispersing females (Fig 4). There was no significant effect of replicate time period on lifetime egg production (ê = 0.230±0.360 (s.e.), *t* = 0.639, *P* = 0.527, n = 42).

## Discussion

Variation in life-history traits in dispersing invertebrates has been well documented, but studies have been skewed towards a few taxa with a strong focus on dispersal by flight. However, it is not necessarily the case that dispersal other than by flight has the same life history consequences. In insects for example, larger individuals can fly and disperse further than smaller individuals which have smaller energy stores [37, 38]. Dispersal by phoresy, however, may eliminate the need for large energy stores and has been suggested as a means to compensate for the disadvantages of small size during long-distance migration [39]. Here we investigated how investing in dispersal affects life-history traits in a mite species that disperses as a deutonymph by phoresy; our results reveal that deutonymph expression correlates negatively with several life history traits of the bulb mites (Table 1).

**Table 1:** Summary of the effects of deutonymph expression.

Trait	Cost/Change	Disperser response (vs. non-dispersers)
**Males**		
**Size at maturity**	Yes	Mature smaller
**Females**		
**Size at maturity**	Yes	Mature smaller
**Lifetime egg production**	Yes	Lower egg production
**Sex ratio**	Yes	Female biased
**Male morph ratio**	Yes	Only fighters (no scramblers)

“Yes” indicates a cost to life-history traits or change to sex or male morph ratio. Disperser response informs of the type of change (if any); e.g. “Mature smaller” indicates dispersers mature at a smaller size than non-dispersers.

Firstly, both male and female dispersers did not compensate for lost growth during the deutonymph stage and matured at a smaller size than non-dispersing individuals. This indicates that expression of a deutonymph stage correlates with a reduced size at maturity, suggesting a cost. It turned out, however, that the length of time that individuals were a deutonymph did not influence their size at maturity, suggesting that any costs associated with being a deutonymph do not accumulate to negatively affect size at maturity. Neither was deutonymph size a predictor of size at which an individual matured. The most likely reason for the reduced size at maturity, of dispersers, may be a combination of factors. Producing deutonymphs has an energetic cost (see [14] for examples of costs of producing dispersal morphology) and therefore requires resources which would ultimately be used for growth. Another factor could be due to the biology of the deutonymph stage itself; this stage is non-feeding and so cannot grow or acquire resources that can be used for growth in a later stage.

Secondly, as in many other taxa [11], we found that the expression of this costly dispersal morphology correlates with reduced lifetime reproductive success suggesting a trade-off between dispersal expression and investment into reproductive success. In the case of longer-lived females (lifespan > 30 days), non-dispersers produced more offspring in their lifetime than did dispersers of the same age. It appears that both sexes are unable to compensate for the energetic investment in producing a deutonymph (inferred from the fact that both sexes show reduced size at maturity and females suffer reduced lifetime egg production).

We also found a correlation between sex ratio and dispersers, and male morph ratio and dispersers: dispersers have a female-biased sex ratio and, more significantly, no scramblers developed from a deutonymph (see also [40] for the same result). Since the sex ratio in the bulb mite is genetically determined [41], ecological factors such as differential survival, probably caused the sex ratio to deviate from 50:50. For example, males might have a lower survival rate than females if they have been a deutonymph, or, although less likely, male deutonymphs on a developmental path to become a scrambler do not survive to adulthood. A more probably explanation for why only male deutonymphs develop into fighters in our study is that their weapons, i.e. their fighter legs, can be a useful tool when arriving in a new environment. In addition to fending off other males when competing for females, fighters can also use their legs to kill and consume other (con- or heterospecific) mites (e.g. [31], [42]) or to defend themselves from predatory mites (Iza Lesna, personal communication). Unravelling why, so far, all male deutonymphs in our study, albeit a correlative study, only develop into fighters adds to the complexity of male morph determination [22]. Fighter expression is environmentally but also in part genetically determined [43]. Given this genetic influence, it remains to be investigated to what extent successful colonisation of a new environment affects the evolution and coexistence of fighters and scramblers if the founder males of a colonising population are all fighters.

Our results show that there is a negative correlation between life-history traits and dispersal and, in males, is male morph specific which is in line with the increasing view that dispersal and life history patterns are interrelated in a complex manner [38]. Additionally, the incurred costs are not always comparable to other modes of dispersal. This can be seen by comparing incurred costs we find in this study to costs incurred by dispersal via flight (Table 2). Life history trajectories are strongly influenced by environmental change [27] and how changes in the environment influence life history patterns in relation to dispersal is another challenge remaining to be addressed. Only by investigating a wide variety of taxa and dispersal modes across a broad range of environmental conditions can we gain a better understanding of the biology and evolution of species life-histories.

**Table 2:** Summary of the comparison between the effects of deutonymph expression (dispersal by phoresy) and dispersal via flight on life-history traits and sex ratio.

Trait	Disperser response (vs. non-dispersers)^§^	Dispersal by flight (vs. no dispersal/short distance dispersal)
**Males**		
**Size at maturity**	Mature smaller	Mature larger^1^ ^#^
**Females**		
**Size at maturity**	Mature smaller	Mature larger^1^ ^#^ ^,^ ^2^ * ^,^ ^3^
**Lifetime egg production**	Lower egg production	Lower fecundity^2^ ^,^ ^4^ ^,^ ^5^
		Higher fecundity^6^ ^,^ ^7^ ^,^ ^8^
**Sex ratio**	Female biased	No sex bias^9^ ^,^ ^10^
		Male biased^11^ ^,^ ^12^
**Male morph ratio**	Only fighters (no scramblers)	NA

^1^Roff [44]

^2^ Dixon et al. [45]

^3^Roff & Fairbairn [46]

^4^Zera and Denno [11]

^5^Bonte et al. [14]

^6^Rankin & Burchsted [10]

^7^Min et al. [12]

^8^Hanski et al. [13]

^9^Fadamiro et al. [47]

^10^Perez-Mendoza et al. [48]

^11^Gäde [49]

^12^Nishigaki & Ohtaki [50]; NA–not applicable

^§^This study

^#^Sexes were not separated in this study

*Measurement was lipid content.

## Supporting Information

S1 Table Post-protonymph growth and survival. Comparison of post-protonymph life stages (tritonymph and adult) between non-dispersers that were collected as eggs or protonymphs.
(DOCX)

S2 Table Full models of life-history trait analyses. Full models including non-significant terms that were removed during the model selection procedure of the life-history trait analyses (p-values in bold indicate significant terms).
(DOCX)
