# Supplementary material for: Life History Consequences of the Facultative Expression of a Dispersal Life Stage in the Phoretic Bulb Mite (Rhizoglyphus robini)
Source: PLoS One. 2015 Sep 1;10(9):e0136872. doi: 10.1371/journal.pone.0136872 (PMC4556651; doi:10.1371/journal.pone.0136872)
Supplement: S1 Table — Comparison of post-protonymph life stages (tritonymph and adult) between non-dispersers that were collected as eggs or protonymphs. (DOCX) [file pone.0136872.s001.docx]

**S1 Table.** **Post**-**protonymph growth and survival**. Comparison of post-protonymph life stages (tritonymph and adult) between non-dispersers that were collected as eggs or protonymphs.

|  |  | **Estimate** | **Std. error** | ***t*-value** | ***z*-value** | ***p*-value** |
| --- | --- | --- | --- | --- | --- | --- |
| Growth* | Tritonymph | -0.0019 | 0.0043 | -0.45 | NA | >0.05 |
|  | Adult | -0.0093 | 0.0058 | -1.6 | NA | >0.05 |
|  |  |  |  |  |  |  |
| Survival† | Tritonymph | 0.001 | 0.2099 | NA | 0.005 | 0.996 |
|  | Adult | -0.1349 | 0.2093 | NA | -0.6044 | 0.519 |

*Analysed using linear mixed effects model with a Gaussian error structure

†Analysed using generalized mixed effects model with a binomial error structure
