# Supplementary material for: Life History Consequences of the Facultative Expression of a Dispersal Life Stage in the Phoretic Bulb Mite (Rhizoglyphus robini)
Source: PLoS One. 2015 Sep 1;10(9):e0136872. doi: 10.1371/journal.pone.0136872 (PMC4556651; doi:10.1371/journal.pone.0136872)
Supplement: S2 Table — Full models including non-significant terms that were removed during the model selection procedure of the life-history trait analyses (p-values in bold indicate significant terms). (DOCX) [file pone.0136872.s002.docx]

**S2 Table.** **Full models of life-history trait analyses.** Full models including non-significant terms that were removed during the model selection procedure of the life-history trait analyses (p-values in bold indicate significant terms).

| **Trait** | | **Estimate** | **Std. Error** | **t-value** | **P - value** |
| --- | --- | --- | --- | --- | --- |
|  |  |  |  |  |  |
| **Size at maturity of dispersers** | |  |  |  |  |
|  | Intercept | 0.627 | 0.014 | 45.214 | **<0.001** |
|  | Sex | -0.115 | 0.026 | -4.378 | **<0.001** |
|  | Deutonymph duration* | -0.001 | 0.001 | -0.812 | 0.425 |
|  | Deutonymph size* | 0.709 | 0.539 | 1.314 | 0.199 |
|  | Treatment* | 0.003 | 0.015 | 0.233 | 0.817 |
|  | Deut. Duration:Deut. Size* | -0.123 | 0.130 | -0.943 | 0.355 |
|  |  |  |  |  |  |
| Size at maturity | |  |  |  |  |
|  | Intercept | 0.712 | 0.011 | 67.464 | **<0.001** |
|  | Sex | -0.130 | 0.014 | -9.114 | **<0.001** |
|  | Deutonymph expression | -0.080 | 0.015 | -5.376 | **<0.001** |
|  | Treatment* | -0.005 | 0.004 | -1.203 | 0.232 |
|  | Sex:Deutonymph expression* | 0.019 | 0.032 | 0.589 | 0.557 |
|  |  |  |  |  |  |
| Lifespan | |  |  |  |  |
|  | Intercept | -1.263 | 1.538 | -0.821 | 0.417 |
|  | Lifespan | 0.485 | 0.057 | 8.444 | **< 0.001** |
|  | Deutonymph | 7.135 | 2.501 | 2.852 | **0.007** |
|  | Lifespan:Deuto. expression | -0.276 | 0.081 | -3.413 | **0.002** |
|  | Treatment* | 0.230 | 0.360 | 0.639 | 0.527 |
|  |  |  |  |  |  |

* Values of terms that were not significant and removed (during model selection) are shown, however the p-value is an indication of the ANOVA comparison between the model including and the model excluding the term indicated.
